# In Their Voices: Client and Staff Perceptions of the Physical and Social Environments of Adult Day Services Centers in Taiwan

**DOI:** 10.1155/2018/5130472

**Published:** 2018-07-29

**Authors:** Chih-ling Liou, Shannon Jarrott

**Affiliations:** ^1^Department of Human Development and Family Studies, Kent State University at Stark, North Canton, OH 44721, USA; ^2^College of Social Work, Ohio State University, Columbus, OH 43210, USA

## Abstract

Studies have examined the impact of environments on long-term care residents' quality of life; however, environment gets little attention in adult day services (ADS). The current study gives voice to clients and staff by capturing their perceptions of the physical and social environments of their ADS centers. Data were collected from 23 interviews with staff and clients and 270 hours of participant observations at two ADS centers in Taiwan. The authors triangulated field notes with interview transcriptions and analyzed them with the Grounded Theory coding procedure method. Findings reveal clients' and staff members' perceptions of appropriate and inappropriate physical and social environmental features affecting quality of life at the center and reflecting Taiwanese culture. We address how perceived appropriate features can be sustained or replicated and how perceived inappropriate influences can be remedied. Results can be translated into action research by implementing supportive environments for both staff and clients at ADS centers.

## 1. Introduction

Adult day service (ADS) is a popular community-based service designed to provide respite to family caregivers by offering long-term care (LTC) services during the day to adults with physical and/or cognitive impairment who need supervised care [1]. ADS programs have rapidly grown in the United States as families and researchers find them beneficial, cost-effective alternatives to nursing homes [1, 2]. ADS programs in Taiwan are largely informed by American models and have also grown drastically because of the government's promotion and planning to develop more centers for the rapid growth of people aged 65 and over: from 10.9% in 2010 to 41.6% by 2060 [3].

ADS researchers have focused primarily on caregiver outcomes of ADS use [4]; attention should now turn to ADS clients and staff who spend most of their day in the care environment. The environment in which LTC is provided consists of a physical and a social environment. The physical environment encompasses the setting, décor, and private spaces, while facility regulations, activities, culture, and interpersonal interactions comprise the social environment [5]. Both the physical and social environments have been closely associated with client well-being [6]. As a “partial institution” where clients do not live together but receive care services similar to those provided at LTC, researchers, practitioners, and families should be concerned with the impact of the ADS environment as well.

ADS clients are particularly susceptible to environmental influences given associated decline in physical and cognitive functioning [7]. The environment can be a therapeutic resource to reduce need-driven behaviors and promote the well-being of persons with dementia [8]. However, it can also contribute to ill-being [5, 9]. Among the limited research on the ADS environment, Lyman [10] discovered that ADS staff responded to increased caregiving demands by exerting increasing control over clients within a physical environment characterized by architectural barriers (e.g., no wheelchair accessibility) and space limitations (e.g., no separate activity rooms). Salari and Rich [9] found that a classroom-like environment encouraged a teacher-student relationship that supported staff tendencies to control clients' behavior. Liou and Jarrott [11] reported that staff working in a hospital-like environment used a nurse-patient style of interaction that focused on physical care and ignored clients' social care. In contrast, a person-centered approach has frequently achieved a therapeutic environment for persons with dementia [6]. Examining the care environment from the perspective of ADS clients can support achievement of a therapeutic physical and social environment.

While models of environmental influence on function, such as Lawton's environmental press model [12], have been developed for elders in LTC, they can also be applied to staff members working in these environments. Staff have intimate interactions with clients and both influence and are influenced by the environments in which these interactions occur [7]. If staff experience demands exceeding their capabilities, such as working with a high client load or low supervision, they may engage in maladaptive behaviors, such as making derogatory comments about clients' abilities [13].

The current study was part of a larger project named “Examination of Social and Physical Environments in Two Centers in Taiwan” possessing two unique features. First, the lead author collected the data at two ADS centers in her native country of Taiwan; her familiarity with the culture and tradition of Taiwan provided a nonwestern cultural lens to data interpretation. Second, we gave voice to both ADS clients and staff who were observed and interviewed for their experiences and perceptions of the centers' physical and social environments. We sought to answer the question:* How do staff and clients at ADS centers in Taiwan perceive the centers' physical and social environments? *We will address those physical and social features perceived as appropriate or inappropriate by ADS staff and clients and recommend strategies for the ADS environment to enhance the quality of life and quality of care at centers.

## 2. Method

We utilized focused ethnography to elicit the perceptions, meanings, and experiences of participants and to provide rich descriptions [14]. Focused ethnographic studies, which are common in nursing research [15], are shorter in nature in comparison to traditional ethnographic study design; however, they still provide in-depth understanding of specific groups and places through interviews and intensive participant observations [14]. Ethnographic studies normally focus on one setting or a small number of settings that are geographically close to the researcher [16]. We had the advantage of doing research at the first author's hometown to develop understanding of the ADS environments' impact reflected in participants' voices [17].

### 2.1. Settings

According to Taiwanese governing regulations, the Department of Social Affairs (DSA) oversees community-based services, and the Department of Health (DOH) oversees institutional services providing medical treatment, located primarily in hospitals and nursing homes [18]. Two centers were selected because they were well known within their respective systems. Center A was supervised by the DSA; it was designed for people with dementia (33% mild, 56% moderate, and 11% severe) with physical features that elicit a sense of clients' past. Center B was operated by a university hospital and regulated by the DOH. Most Center B clients (83%) had cognitive impairment. Both centers, regulated by different departments, were promoted by the government as providing community-based services meeting the clients and family caregiver needs.

### 2.2. Participants

Participants included ADS clients, staff, and volunteers. There were between 28 and 34 clients in Center A when this study was conducted. Most of the clients attended the center every weekday, and five or so came two to three times a week. The female clients were the majority, accounting for 65% of the total group. The average age of clients in Center A was 80. More than half of the clients were widows or widowers and lived with their adult children, particularly their sons.

There were between 17 and 19 clients who attended Center B daily. The number of female and male clients was almost even in Center B, where 58% were female and 42% were male. The average age of the clients in Center B was 80. Like Center A, more than half of the clients were widows or widowers and lived with their adult children, with sons in particular.

Including both full- and part-time staff, there were 11 employees in Center A: one director, one nurse, five nurse aides, one social worker, two bus drivers, and one cook.

Staff members in Center B were one director, one nurse, three nurse aides, one bus driver, and one housekeeper. There were two to three regular volunteers at Center B every weekday, whereas no volunteers attended Center A regularly. Volunteers were included because they also contributed to the social environments and influenced clients' behavior.

### 2.3. Data Collection

Data were collected through participant observation, interviews, and examination of related documents, which characterize most ethnographic research [19]. After Institutional Review Board approval, the first author made participant observations at both centers for eight hours a day, five days per week for a total of 240 hours. The observer also served as a volunteer to support staff and clients during programming. Volunteering allowed the observer to build a close relationship with clients and staff. Clients and staff became more open over time and shared their thoughts with the observer, enabling her to critically compare individuals' statements with her observations.

The first author conducted semistructured interviews with eight clients, one volunteer, and 14 staff. For the clients, they were asked about their lives in the center, feelings about the activities, circumstances surrounding their attendance, and relationships with staff. Staff and one volunteer were interviewed on their perceptions of clients, their views on their own roles at center, and what they think about the environment of the center. All interviews were conducted and audio-recorded in private space at the centers and lasted from 40 to 90 minutes. Clients and staff described their thoughts on the physical and social environments at the center. Interviews were conducted in Mandarin Chinese, transcribed in Mandarin Chinese, and then translated to English by the first author.

### 2.4. Data Analysis

We selected the Grounded Theory (GT) coding method because it takes researchers into the real world so that findings emerge from participants' voices [20]. GT analysis began when all of the interviews and field notes were concluded and completely transcribed. The first author independently open-coded all transcriptions line-by-line, which Charmaz [21] recommended for ethnographic research. Altlas.ti Version 7 was utilized to facilitate data coding. The initial open coding was followed by focused coding to select some initial codes, which make the most analytical sense based on our research questions. Next, 1062 initial codes were combined into 17 themes (see Table 1); axial coding involved a process of reviewing the themes and placing them into subcategories around the phenomena of care environment. Previous ADS ethnographic studies examining the environment used structured subcategories to do the axial coding [5, 22, 23]. To bring coherence to the analysis and identify patterns to inform environment-enhancing strategies, we structured subcategories with a simple distinction between appropriate and inappropriate physical and social features (see Table 1). Appropriate features reflected a positive evaluation of the feature or its impact on participants or staff in the center. Inappropriate features were evaluated negatively for their impact on center occupants.

## 3. Results

### 3.1. Clients' Perceptions of the Physical Environment

During the interviews, clients were asked about their opinions on the setting, décor, and other physical characteristics of the center that comprise the physical environment. Overall, they either expressed little interest in talking about the physical environment or indicated that the physical environment exerted little influence on them.

Clients responded, “I have nothing to share,” or “We human beings will get used to a new [physical] environment if we stay there for a while, so we have gotten used to everything within this [physical] environment.” Although the clients did not want to directly comment on the physical features during the interview, they talked more about their preferences and dislikes of this environment during informal conversations.

#### 3.1.1. Appropriate Physical Features

Information from the informal conversations revealed that the clients in Center A liked its* old-style setting* and* cleanliness*. Center A was originally designed and built for people with dementia to elicit a sense of their past. It created an atmosphere of an old-fashioned Taiwanese living space familiar to people who are now in their 70s to 90s. From the main entrance to the restrooms and the décor in the living room, interior features were designed with a reminiscent orientation to a 1960s and 1970s' Taiwanese home or community. More than half of the clients at Center A shared the fact that “the old style design helps us to talk more and creates a feeling of belonging in the center.” “Here it is just like the place I grew up. I feel secure here.” They rated the physical environment as “very clean” so they “feel safe here.” One male client interpreted cleanliness as a sign that “it is not for people who are insane or poor,” which reflects a Taiwanese stereotype that houses for poor, homeless, or mentally disabled people are unclean. Therefore, clients appreciated feeling safe in a clean center because they did not encounter people who are mentally ill or poor.

#### 3.1.2. Inappropriate Physical Feature

Center B's clients complained the* center's small size* so they could not find a place to rest privately or “cannot walk easily in the center.” Interestingly, by the end of the conversation, they all told the first author that it is not their business to talk about the physical environment at the center because “this is not my home” or “we are here as the guests not the masters.”

Lawton [12] stated that sometimes people do not pay attention to their physical environment or are unaware of its influence on them. In contrast, clients in this study not only paid attention to the physical environment but also were aware of its influence on their lives at the center. They might have hesitated to speak directly about the inappropriate physical features because they felt powerless to change them. Our data suggest that caretakers should not automatically assume that the physical environment meets all the clients' needs if they do not voice complaints.

### 3.2. Staff Members' Perceptions of the Physical Environment

Unlike the clients, the staff at both centers willingly shared their opinions of the influence of the physical environment on them and their clients during both formal interviews and informal conversations. Staff identified appropriate and inappropriate physical environmental characteristics that affected clients and their ability to care for them.

#### 3.2.1. Appropriate Physical Features

Staff perceived that appropriate physical features reflected* a client-centered interior design* and* open space layout*. Center A staff described the old-style design as client-centered because “this old-style setting is copied from the environment in which they [the clients] grew up. When they [the clients] are in this old-style environment, they feel familiar and safe, and easily accept staff directions.” That is, the interior design created a therapeutic atmosphere to help clients remember and talk more about the past. Moreover, the familiarity relieved clients' anxiety, leading to fewer behavioral problems. Additionally, the open design kept sight lines clear so staff could easily find where the clients were at the center. If the center was not designed as an open space, it would have had partitions blocking staff members' view of clients, thereby increasing supervision demands. As a result, staff workload and stress were low, and staff were able to concentrate on activities and positive interactions with the clients.

The open space design offered advantages but also had unwanted effects. Staff reported one downside of the open space design: “the clients with moderate or severe dementia are easily distracted because the noise from other groups travels across the center and influences the clients.” To solve this problem, staff suggested curtains at the center would absorb sound and create the perception of closed space.

#### 3.2.2. Inappropriate Physical Features

Inappropriate physical features perceived by staff included features of* a non-client-centered interior design*,* small size*, and* limited seating*. The first two features presented in Center B, which was designed as an emergency room. Because of its appearance, staff reported that “the newcomers with moderate/severe dementia feel insecure at the center and repeatedly ask to go home.” Repeated efforts by staff to convey that clients were not in a hospital failed; newcomers still associated Center B with a hospital and continued to search for an exit. The physical space of Center B further challenged staff work because wall placement created blind spots where the staff and clients could not see each other. The combination of wandering clients and blind spots required high staff supervision. In response, staff restrained clients at risk of falling in wheelchairs.

Like the clients at Center B, staff also complained about its small space. The nurse described the dining area as it “is barely able to accommodate half of the clients - the small dining room increases the risk of falling.” The director shared that three clients had fallen in the previous month; consequently, she required the staff to supervise the clients at all times. Staff agreed that Center B requires remodeling, such as removing some walls and painting the walls with a warm color to make the space more accessible, safe, and inviting. Unfortunately, staff expressed little hope of remodeling: “It is hard to make any changes here…the administrators do not pay attention to the physical environment.”

Although Center A staff and clients alike complimented the physical environment, there is one inappropriate element; insufficient seating in the living room led clients to fight for a seat. The social worker at Center A suggested that clients claimed chairs as theirs to form a sense of belonging. When clients could not find a chair in the living room, a disrupted sense of belonging might have contributed to restless behavior.

### 3.3. Clients' Perceptions of the Social Environment

#### 3.3.1. Appropriate Social Features

Appropriate social features described by all clients in the interviews encompassed* being treated with respect, being treated like family,* and* having someone to watch over them*. Based on Confucianism, which emphasizes respect for age and seniority, the appropriate treatment of old clients involves showing them respect [3]. Participants in this study were happy to be greeted as “grandma” or “grandpa” with a sincere manner. In Taiwan, calling someone aged 65 and over “grandpa” or “grandma” is a way to respect and honor their life experiences even though they are not biologically related.

During activities, staff at Center A did not view clients as passive participants but as contributors or teachers, telling the clients, “I learned a lot from you and will remember what you taught me today.” Responding to client feedback was another common form of showing respect to clients. Most of the time, staff responded to clients' questions promptly, just as clients expected to be treated at home.

In addition to being treated with respect, clients valued kin-like relationships with staff members. One female client stated that “the staff here are thoughtful and concerned about me and treat me just like family.” The majority of clients said that they were alone and bored at home and enjoyed being at the center, including one client who “wished to stay at the center 24 hours each day.” Although staff were perceived occasionally as “bossy,” clients tolerated directives when they felt that the staff treated them like family. A good quality of life for Taiwanese ADS clients includes respectful treatment reflecting family dynamics common among Taiwanese families.

Some clients, however, did not care whether they were treated like family but were merely contented with having someone to care for them while their adult children worked. One female client shared the fact that “after falling at home several times…my daughter brought me here to have more people watch over me. The best thing about coming here is having someone to take care of me.” Some clients tolerated staff's inappropriate treatment so long as they received supervision, which eased their adult children's worries.

#### 3.3.2. Inappropriate Social Features

Inappropriate social features named by the clients at both centers were embodied by the* collective life at the center*. Clients were aware of the bureaucratic management that shaped their lives at the centers. When asked about invitations from staff to join the activity, one client responded passively: “I am not “invited” but assembled. They just announce, ‘Go to the tea shop,' so I know where to go and what to do.” Such a response echoes Goffman's [24] characterization of the total institution in which individuals were forced to forego self-determination, autonomy, and freedom of action to meet staff expectations.

Some clients, however, embraced the uniformity: “Here [The center] is for a group of people to do the same things together for the same goal.” According to Goffman [24], when institutional goals did not match individual needs, most individuals might find a way to justify their compliance with institutional rules. One client, a veteran, described during the interview, “I just follow whatever they [the staff] tell us…. I have been used to this kind of life since I was in the army.” The army is an example of a total institution in which people follow all orders, live the same schedules, and give up self-authority in order to increase technical skills [24].

Other clients shared the fact that “being at the center is just like being at school. The staff are the teachers so we have to ask them where to go and what to do if we do not know.” The Taiwanese cultural tradition stresses hierarchical relationships and collectivism in which individuals surrender autonomy to a higher authority in order to reach a group goal [25]. In Taiwan, four types of occupations have authority over others, and teachers are one of them. Therefore, some clients did not just passively tolerate the staff's direction but believed that they as “students” were supposed to obey staff as “teachers.”

### 3.4. Staff Members' Perception of the Social Environment

#### 3.4.1. Appropriate Social Features

Staff identified* a sense of collective life* and* emotional suppression *as appropriate social features. Staff agreed that collectivism is the best way to orient the facility because “if the clients just do whatever they want at the center, they will break the rules and make things difficult.” Following a practice of collectivism, similar to total institutions, means that “they [clients] have to follow our directions while doing things. Freedom might be the thing that elders here do not have.” The collectivist approach applied to staff as well as clients. One staff member shared the fact that “everyone here has to follow a schedule of things to do, and that is same for the staff who also follow the schedule to lead the activities.”

The collective life at Center A was evident during activities, when staff provided one-way, call-and-response instruction. Every client had to follow the staff member's direction; even sleep was prohibited during the activity. Staff believed that their responsibilities included leading group activities that engaged every client. The Center A director indicated that effective activities “keep their [clients'] attention and reduce their dementia-related behaviors. Because of participating in the activities, the elders here have fewer behavior problems than they have at home.” In order to lead a successful group activity, Center A staff treated clients like school students, for example, asking them to answer questions with a loud voice or to study hard at home for activities at the center. Staff believed that this hierarchical, teacher-student communication style promoted engagement and helped clients use their brain to slow cognitive decline.

To “protect” the clients, staff at Center B compelled all clients to exercise and directed them on how to eat their food. One afternoon, a nurse's aide led exercises. She scolded a female client for not doing the exercises, explaining to the group that the woman's illness resulted from inactivity. Volunteers followed suit, directing the woman to perform the exercises; one said “some clients are really lazy so we have to push them to do some exercise to move their body to keep them healthy.” The staff and volunteers did not ask the client why she did not participate but concluded that she was lazy. This conclusion reflected a collectivist approach because the client did not follow the group.

Directing the clients on how and what to eat was viewed as another way to “keep clients healthy” at Center B. During lunch time, aides and volunteers walked around the dining tables pushing clients to finish their food. The director of Center B claimed that the clients would not otherwise eat vegetables. When staff help clients put the vegetables in their bowls and “tell them that the vegetable is good for their health, the elders eat them all.” Aides also controlled clients' pace of eating. To have all clients napping at the same time, aides rushed clients to finish, saying “you have to eat fast and finish your food as soon as possible. Otherwise, it will be too late for you to use the restroom, so you may pee your pants again.” The staff explained that they cared more about clients' physical health because if they did not take good care of the clients physically, they would not be able care for them psychologically.

Consistent with family caregiver studies [4], staff reported that caring for clients with dementia was emotionally demanding and that they had to suppress their emotional reactions. Despite their efforts to hide emotions, staff admitted that their emotions were sometimes affected by the clients. One staff member confessed that she easily lost patience; when that happened, other staff members reminded her to calm down and helped her distract the clients: “I have worked here for two years and been asked those same questions million times. Even though I know that their behavior is driven by their disease, I am still influenced by their behavior and feel frustrated and lose patience.” If the staff sensed themselves being negatively influenced by the clients, the appropriate response was leaving the situation and having other staff assume responsibility. To maintain good mental health, one staff member reported “we have to know how to adjust our minds and release our tensions. If we accumulate too many negative emotions, it will lead to bad interactions with the elders.”

#### 3.4.2. Inappropriate Social Features

Staff did not directly recognize the inappropriate social features at center, but the lead author observed some included* confining clients in wheelchairs* and* labeling them*. Staff at Center B admitted they knew confinement was inappropriate, but they “do not know other ways to deal with clients' challenging behavior.” One female client with severe dementia was capable of walking alone yet was strapped in a wheelchair every day. The nurse explained: “That client has a risk of falling due to her medication [to control her aggressive behavior]. In order to prevent her falling, we have to confine her.” However, the female client was not satisfied with being confined in a wheelchair and struggled to get out of the wheelchair by taking off all her clothes. The confinement intended to reduce one undesirable behavior actually triggered another behavior, but staff preferred she struggle in a wheelchair than let her move around freely and risk of falling.

Staff at both centers labeled the clients with their diagnosis and attributed the clients' challenging behaviors to their understanding of the diagnosis, which was not always accurate, as illustrated by the staff member who said, “We do not know when they [the clients] will go crazy…. The only thing I can do is to tell myself that they are sick.” Staff members associated clients' challenging behavior with a medical label rather than linking the behavior to a need that staff members might be able to address. The clients at both centers responded to staff's labeling by colonizing the idea and labeling themselves or other clients as persons who “will become senile,” “have a childish mind,” and “are similar to children.” According to labeling theory, clients' self-labeling and the staff's labeling gave staff members power to control client behavior [26].

Reflecting the power dynamic of the two ADS centers, staff members forced rules upon clients who were expected to be physically and cognitively incompetent. Client acceptance of staff labeling or peer self-labeling can be understood through a cultural lens. Clients recognized the staff as authority figures in Taiwanese society and submitted to caregiving practices that detracted from client personhood even when treated like family.

## 4. Discussion

Environment gets little attention in LTC outside nursing homes, though studies highlight its influence on the behavior and attitudes of staff and clients [12]. Little is known about clients' and staff members' perceptions of the ADS environment, which is unfortunate as clients with cognitive and functional impairment associated with dementia are especially vulnerable to environmental influences [7]. Our study provides an in-depth understanding of two ADS centers from both staff and client perspectives with a cultural consideration.

Previous research demonstrated that appropriate physical environmental settings reduced accidents and problem behaviors and enhanced occupants' wellness [7]. In contrast, poorly designed environments limit clients' mobility and independence, negatively affecting their health and behavior. In the United States, a home-like interior design has been promoted to benefit nursing home residents [27]. In Taiwan, we demonstrated that the client-centered interior design is much more than a place that looks like home with noninstitutional furniture; rather, its features matching clients' temporal experiences. Clients receiving care in this matched environment quickly adapted to its physical environment, experienced belonging and security, conversed comfortably with peers, and engaged in fewer aggressive behaviors. Staff also benefited from working in the client-centered physical environment of Center A, reporting low levels of stress managing client behaviors and high levels of energy for personal interactions with clients. The physical features enhanced both clients' and staff members' quality of life at the center, which aligns with Kitwood's [28] conceptualization of person-centered care that creates a more positive experience of life for staff and clients.

In contrast, the interior of Center B, reflecting its past use as an emergency room, made new clients uncomfortable and led to challenging behaviors, such as wandering. The staff faced significant demand supervising wandering clients, even confining some to wheelchairs to keep them stationery. The effect of the unsupportive physical environment at the center contributed to a restless social environment, as Altman [29] argued in his social systems model depicting bidirection influence between behavior and environment. In the present study, this model is illustrated by a feedback loop in which the physical environment influenced staff care practices, which shaped client response to the care setting and, ultimately, affected staff response to clients.

A home-like physical environment is most valuable to occupants when it corresponds to a home-like social environment [8]. A home-like physical environment promotes clients' desire for independence. However, if the social environment entails strict schedules and inflexible practices that silence clients' independence, the therapeutic potential is undermined, and maladaptive behaviors are common. In the current study, we found that the supportive physical environment at Center A led to both positive and negative outcomes. On the one hand, the client-centered interior design created a home-like atmosphere to encourage the clients to exercise control, which could enhance their self-esteem [27]. Clients in Center A cared about their rights and benefits and fought with staff and other clients to exercise their authority. On the other hand, the home-like atmosphere fostered a close, quasi-kin relationship where staff viewed the clients as their parents or grandparents. This kin-like relationship eroded the clients' autonomy when staff demonstrated their respect to clients by doing things or making decisions for them, as they would serve their aging parents at home [11]. Staff members' respectful intentions actually infantilized the clients, fostered learned helplessness, and contributed to a negative self-image.

In contrast, within Center B, clients usually claimed that they were hospitalized rather than in a “home.” Staff shared in interviews that clients' families also associated the ADS with a hospital. The perception of Center B as a hospital may be interpreted through the cultural lens of the traditional Confucian value of filial piety. Fifteen elements comprise classical Chinese Confucianism; four of them emphasize how to serve, treat, and look after one's parents [30]. Accordingly, adult children are responsible for providing good care to their aging parents at home with a spirit of true caring [31]. Leaving a parent at ADS may feel like a violation of a child's duty [32]. To protect themselves from feeling guilty, adult children may adopt the idea that Center B is like a hospital; they can then justify their decision to seek ADS services as a treatment for their parents, rather than a failure to practice filial piety.

The notion of being in a hospital led new clients to feel insecure, while long-term clients possessed low expectations for social and emotional support from the staff. Most Center B clients limited their requests or complaints. They silently accepted their care and attributed their tolerance of inappropriate treatment to a value for compromising one's needs for a common good. Compromise is embedded in Taiwanese culture where harmony and collectivism are emphasized. Taiwanese people are taught that self-suppression is valued over self-expression, especially in institutional settings [32]. If something or someone might damage the harmony of the institution, the Taiwanese will compromise to maintain a calm collective. The institutional physical setting of Center B fostered harmony by suppressing the voices of clients seeking to avoid conflict with providers respected as medical professionals [31]. Professional caregivers can benefit from knowing if their clients grew up in a collectivist culture. These clients may be appreciated as “good” clients with few complaints who are easy to care for. Culturally appropriate techniques may be needed to elicit clients' preferences if they diverge from the collective. Further, these clients may face greater risk of mistreatment in semi-institutional care settings as they have learned to sacrifice their preferences for a predominant group good.

Collective life was the dominant theme in our study. The lives of both clients and staff were dictated almost entirely by a fixed schedule with frequently repeated activities that limited choices or control for either group. Caregiving focused heavily on the group as individual needs were put aside. Without any choice or control over everyday matters at the center, some clients, particularly those at Center B, felt unvalued and expressed low self-esteem. Staff at such sites can learn to offer clients more decision-making opportunities while maintaining their safety and respecting their cultural backgrounds [32].

Just as direct care staff are advised to support a positive social environment among clients, their administrators should also foster a social environment that promotes staff well-being [13]. Staff choices at both centers were limited as they enforced collectivism at the behest of care management; studies in Taiwanese nursing homes yielded similar results [32, 33]. Though our data were gathered in Taiwan, other studies suggest that ADS in other countries without collectivist traditions also often hold expectations that participants will join programming in a collectivist fashion [5]. Future care professionals at ADS centers may find value in attending to clients' expectations of the program. They may consider shifting from traditional, collectivist attitudes and behaviors to a person-centered model with participant-driven programming, as well as incorporating clients' interests, preference, and abilities into care delivery to optimally support individuals' autonomy, competence, and relatedness.

## 5. Limitations and Future Directions 

We based our study on rich data from observations and interviews with the clients and staff (including volunteers) at two ADS centers in Taiwan. This study is one of the few ADS studies that derived findings directly from the clients' and staff's perception. Our findings reflect how deeply the physical and social environments affected clients' quality of life and staff's care delivery centers in care settings. Although data gathered at two centers cannot be considered representative of all Taiwanese ADS environments, the results can inform future research on other ADS centers. To capture a holistic view of individual differences, future research should include a third group of informants [34]—clients' family caregivers—to more fully understand individuals' experiences.

We conducted interviews with those able to hold coherent conversations; clients with advanced dementia were observed but excluded from interviews. Future studies of the ADS environment may seek additional methods to represent the experiences of ADS clients with greater cognitive impairment. For example, Carroll and colleagues [34] posed closed-ended questions to cognitively impaired clients on topics such as environment, food, safety, activities, autonomy, and socializing. Dementia Care Mapping yields a detailed behavioral map of participants' behavior and affect, including staff behaviors that support and detract from personhood [35]. Efforts to gather information directly from clients can inform practice as well as convey to clients that their opinions are valued. Assessments of occupants' experiences in a built environment can be integrated with architectural assessments of physical features to inform design and remodeling of care facilities [36]. Future aging services providers collaborating with ADS practitioners have the moral and ethical responsibility to provide quality care, in part by ensuring that clients' voices are heard.

## 6. Conclusion

The goal of the current study was to listen directly to ADS clients and staff about their perceptions of the impact of the center's environments. Clients and staff at different environments experienced different daily lives at the centers. At Center A, clients felt secure because the physical environment reflected their life experiences and produced a sense of belonging like what they experienced at home. The home-like environment, however, had both positive and negative impacts on clients. At Center B, both clients and staff disliked the institution-like physical and social environments, which contributed to inappropriate treatment by the staff of the clients. This study revealed important implications on how clients and staff were positively and negatively affected by the physical and social features at ADS centers. Our findings showed that both clients and staff were well aware of the physical environment's influence on their lives at the center but believed they were powerless to change the problematic physical features at their center. The social environment of practicing collectivist traditions at both centers led to the mistreatment of clients by prohibiting them from exercising their autonomy. Previous research has emphasized providing a home-like environment at LTC settings; however, supporting clients' quality of life and improving the center's quality of care, care providers must also consider the social environment as a means to amplify positive outcomes. This study, therefore, lays the foundation for future research focused on enhancing the ADS environment and supporting the quality of life of ADS clients through a cultural lens.

## Figures and Tables

**Table 1:** Initial themes and subcategories reflecting participants' views on ADS environment.

Subcategories	Themes
*Clients' perceptions of the physical environment*	

Appropriate physical features	Old style setting (Center A)
	Cleanliness (Center A)

Inappropriate physical feature	Small, hospital-like space (Center B)

*Staff's perceptions of the physical environment*	

Appropriate physical features	Old style setting matches the clients' life experiences (Center A)
	Open-space layout (Center A)

Inappropriate physical feature	Open-space layout (Center A)
	Limited seating in the living room (Center A)
	Hospital-like environment (Center B)
	Small space with confusing floor patterns (Center B)

*Clients' perceptions of the social environment*	

Appropriate social features	Being treated with respect (Center A)
	Being treated like family (Center A)
	Having someone to watch over me (Center B)

Inappropriate social features	Collectivist orientation neglects individual choice (Center A) (Center B)
	Being at the center is like being at school (Center A)

*Staff's perceptions of the social environment*	

Appropriate social features	A sense of collective life (Center A) (Center B)
	Emotional suppression when working with elders (Center A)

Inappropriate social features	Confining clients in wheelchairs (Center B)
	Labelling the clients (Center A) (Center B)

## Data Availability

Data supporting the results reported in this article can be requested from the first author.
